# The mediating role of emotional intelligence in the relationship between nursing work environment and work engagement among nurses in hematopoietic stem cell transplantation wards: a cross-sectional study

**DOI:** 10.3389/fpsyg.2026.1722783

**Published:** 2026-02-20

**Authors:** Yue Liu, Yingdan Huang, Huifen Wang, Shan Liu, Yaping Bi, Jia Sun, Tingting Liu, Yani Wang

**Affiliations:** 1 Department of Nursing, Hubei Cancer Hospital, Tongji Medical College, Huazhong University of Science and Technology, Wuhan, China; 2 Department of Lymphoma Medicine, Hubei Cancer Hospital, Tongji Medical College, Huazhong University of Science and Technology, Wuhan, China; 3 Department of Hematology, The First Affiliated Hospital of Chongqing Medical University, Chongqing, China; 4 Institute of Hematology and Oncology, Harbin First Hospital, Harbin China; 5 Department of Lymphoma, Chongqing University Cancer Hospital, Chongqing China

**Keywords:** emotional intelligence, hematopoietic stem cell transplantation, nurse, nursing work environment, work engagement

## Abstract

**Background:**

Work engagement is crucial for nursing performance, and the nursing work environment plays a significant role in influencing engagement. Emotional intelligence (EI) has been suggested as a potential mediator in this relationship. This study aimed to evaluate the relationship between the nursing work environment and work engagement, considering the mediating role of emotional intelligence among Chinese nurses in hematopoietic stem cell transplantation wards.

**Methods:**

A cross-sectional design was used. Chinese nurses from hematopoietic stem cell transplantation wards in 19 hospitals with qualifications for hematopoietic stem cell transplantation, located across 9 provinces (or cities), were selected using convenience sampling from February to July 2023. Data were collected through a general information questionnaire, the Nursing Work Environment Scale, the Emotional Intelligence Scale, and the Work Engagement Scale. Structural equation modeling (SEM) was employed to investigate the mediating effect of emotional intelligence between the nursing work environment and work engagement.

**Results:**

Nurses reported moderate levels of nursing work environment, emotional intelligence, and work engagement. The total score of the Work Engagement Scale was positively correlated with the total scores of both the Emotional Intelligence Scale and the Nursing Work Environment Scale, as well as with scores across all dimensions (all *p* < 0.05). Emotional intelligence partially mediated the relationship between the nursing work environment and work engagement, accounting for 24.4% of the total effect.

**Conclusion:**

The findings suggest that both the nursing work environment and emotional intelligence significantly influence work engagement among nurses in hematopoietic stem cell transplantation units. Emotional intelligence plays a key mediating role, which can inform strategies for enhancing work engagement in this setting.

## Introduction

Hematopoietic stem cell transplantation (HSCT) is an established treatment for a range of hematological malignancies and non-malignant disorders (Xu et al., 2018; Duarte et al., 2019). Globally, over 82,000 transplants are performed annually, with growing demand for specialized nursing care (Niederwieser et al., 2022) In China, HSCT activities have expanded rapidly, accounting for 41% of transplants in the Asia-Pacific region (Iida et al., 2023; Xu et al., 2024). However, HSCT nurses face high emotional and physical demands, compounded by staffing shortages and limited career development pathways (Wang et al., 2021). These challenges underscore the need to understand factors that promote work engagement—a positive, fulfilling work-related state characterized by vigor, dedication, and absorption (Schaufeli et al., 2002).

The Job Demands-Resources (JD-R) model provides a robust theoretical framework for understanding work engagement (Demerouti et al., 2001; Bakker and Demerouti, 2007). According to the model, job resources (e.g., supportive work environments) and personal resources (e.g., emotional intelligence) foster engagement through motivational processes. Previous studies in Europe and North America have demonstrated the importance of the nursing work environment and emotional intelligence in predicting engagement among nurses (Shin et al., 2023; Boudreau and Rhéaume, 2024). In Asia, studies from Iran, Japan, and China have also highlighted the role of emotional intelligence in mitigating burnout and enhancing job performance (Wong and Law, 2002; Karimi et al., 2021; Alinejad et al., 2023). For instance, Alinejad et al. (2023) found that EI mediated the relationship between occupational stress and job performance among Iranian nurses, underscoring its role as a crucial personal resource. Karimi et al. (2021) further reported that EI contributed to nurses’ wellbeing and quality of patient care, emphasizing its significance in high-demand settings.

Recent research has increasingly examined the interplay between organizational and personal resources within the JD-R framework. In Europe, Lana et al. (2025) linked a supportive nursing work environment to successful Evidence-based practice (EBP) implementation, urging collaborative efforts between healthcare and academic institutions to foster a theory-driven environment with adequate staffing. In South America, Ruiz-Frutos et al. (2022) reported a strong positive association between work environment and engagement during the COVID-19 pandemic. In North America, Lake (2002) and Wei et al. (2018) emphasized the structural and cultural aspects of the nursing work environment as key drivers of nurse outcomes. In Asia, studies by Zamanzadeh et al. (2013) in Iran and Xia et al. (2016) in China highlighted the intense emotional labor and high workload in HSCT settings, pointing to the need for emotional intelligence training as a buffer against burnout.

However, despite these advances, several gaps remain. First, most studies have focused on general nursing populations, with limited attention to highly specialized and emotionally demanding contexts such as HSCT units. Second, while both nursing work environment and emotional intelligence have been independently linked to work engagement, the mechanism through which organizational resources (e.g., work environment) influence personal resources (e.g., emotional intelligence) to enhance engagement remains underexplored within the JD-R model. Third, although mediation effects have been tested in other nursing contexts—for example, Alinejad et al. (2023) found EI mediated stress-performance relationships—few studies have empirically examined EI as a mediator between nursing work environment and work engagement in high-stakes settings like HSCT. This gap is salient given the extreme emotional and physical demands on HSCT nurses, who face high burnout risk and where emotional competencies may be particularly crucial for sustaining engagement.

Guided by the Job Demands-Resources (JD-R) model, this study aims to explore the mechanisms linking the nursing work environment (an organizational job resource) and work engagement among HSCT nurses, with a specific focus on the mediating role of emotional intelligence (a key personal resource).

The proposed relationships are grounded in both theory and prior empirical findings. Hypothesis 1 (NWE → WE) is supported by consistent evidence across diverse settings showing that supportive work environments enhance engagement. For instance, Ruiz-Frutos et al. (2022) reported a strong positive association during the COVID-19 pandemic, while Lake (2002) and Wei et al. (2018) highlighted the importance of structural and cultural aspects of the NWE. In the context of HSCT, a positive work environment may be especially critical given the high cognitive and emotional demands, suggesting that organizational support can directly buffer against exhaustion and promote vitality and dedication.

Hypothesis 2 (EI → WE) is supported by research identifying emotional intelligence as a critical personal resource that facilitates adaptation and positive work outcomes. Wong and Law (2002) demonstrated its positive impact on job performance and attitudes, and Karimi et al. (2021) linked it to nurses wellbeing and quality of care, underscoring its relevance in demanding contexts like HSCT. Emotional intelligence enables nurses to regulate their own emotions, empathize with patients and colleagues, and navigate interpersonal challenges—all of which can enhance their absorption and dedication to work.

Hypothesis 3 (NWE → EI → WE) extends the JD-R model by proposing that job resources can foster personal resources, which in turn amplify engagement. This aligns with the concept of “resource caravans,” where resources accumulate and interact (Bakker and Demerouti, 2007). Although direct links are established, the integrated pathway wherein the work environment cultivates personal resources like EI remains less explored. However, emerging research hints at this mediating mechanism. For example, Alinejad et al. (2023) found that EI mediated the relationship between occupational stress and job performance, suggesting that personal resources can transmit the influence of environmental factors. In HSCT settings, a supportive environment may provide the psychological safety, supervisory support, and collaborative climate necessary for nurses to develop and exercise emotional skills, which then enhance their engagement.

Therefore, building on the JD-R model and extant literature, we propose the following hypotheses:

*H1*: The nursing work environment is positively associated with work engagement.

*H2*: Emotional intelligence is positively associated with work engagement.

*H3*: Emotional intelligence mediates the positive relationship between the nursing work environment and work engagement.

Testing this integrated model is crucial both theoretically and practically. Theoretically, it extends the JD-R model by elucidating a specific resource pathway (organizational → personal → engagement) in an understudied, high-demand nursing population. Practically, identifying EI as a mediator provides a dual-target intervention strategy: improving the work environment while also enhancing nurses’ emotional competencies.

## Materials and methods

### Design

This study employed a multicenter, cross-sectional survey design using self-report questionnaires administered via an online platform. This design is suitable for describing the status of variables and exploring relationships between them at a specific point in time and is commonly used for testing mediation models.

### Participants

#### Inclusion and exclusion criteria

Clinical nurses working in the HSCT ward were surveyed. The inclusion criteria were as follows: (1) registered nurses; (2) at least 1 year of clinical nursing experience in the HSCT field; (3) provided informed consent and voluntarily participated in the study. Exclusion criteria included: (1) absence from clinical nursing duties for more than 3 months.

#### Sample size analysis

The required sample size for Structural Equation Modeling (SEM) in this study was calculated based on the rule of thumb from Tinsley and Tinsley, which suggests a participant-to-variable ratio of 5–10 (Tinsley and Tinsley, 1987). The total number of measured variables was 57 (16+15+26), which would require a sample size of 330–660. Considering a 20% loss to follow-up, a minimum sample size of 396 is needed. Additionally, according to Schumacher and Lomax (2015), sample sizes in SEM studies can range from 100 to 500 or more, depending on the study design.

#### Hospital selection and data collection

This study used a convenience sampling method. Participants were recruited from 19 hospitals accredited for HSCT across nine provinces/cities in China (e.g., Hubei, Hunan, Anhui, Sichuan, Heilongjiang, Liaoning, Chongqing). Hospitals were selected based on the following criteria: (1) possession of a national health commission-accredited HSCT program; (2) being a tertiary A-grade hospital; (3) performing ≥ 50 HSCT procedures annually. While this multicenter approach enhances diversity, it is acknowledged that clustering effects at the hospital level (e.g., due to shared management systems or institutional culture) may exist and were not controlled for in this analysis, representing a limitation for future research to address using multilevel modeling.

Data collection took place between February and July 2023. A total of 550 questionnaires were distributed. Finally, 523 completed questionnaires were returned, yielding a response rate of 95.1%. After excluding incomplete responses, data from 510 nurses (97.51% of returned questionnaires) were analyzed. Permission was granted by the nurse managers. The questionnaires were distributed using Questionnaire Star, an electronic platform. The link to the electronic questionnaire was sent to ICU nurse managers via WeChat, who then distributed it to the nurses. A standardized introduction was provided to explain the study’s objectives, significance, and completion instructions.

Participants could complete the questionnaire anonymously by scanning a QR code. To ensure data integrity, responses were limited to one submission per IP address. All items were marked as required responses, and results were automatically captured in the backend. Only fully completed questionnaires without logical errors were included in the analysis.

#### Instruments

General Information Questionnaire: A self-constructed instrument designed to collect demographic data, including gender, age, professional title, position, educational qualifications, years of experience in transplant nursing, marital status, nature of employment, night shift frequency, and monthly income.

##### Utrecht work engagement scale

This study employed the Chinese version of the UWES, originally developed by Schaufeli et al. (2002) and translated and adapted by Zhang and Gan (2005). The scale consists of three dimensions—vigor, dedication, and absorption—comprising a total of 15 items. Example items include: “At my work, I feel bursting with energy” (vigor), “I am enthusiastic about my job” (dedication), and “I am immersed in my work” (absorption). Responses were rated on a 7-point Likert scale, ranging from 0 (never) to 6 (every day), with a total score range from 0 to 90, where higher scores indicate greater work engagement. Scores were categorized as “low” (mean ≤ 2), “moderate” (2 and < 4), and “high” ( ≥ 4). The Cronbach’s α coefficient for this scale in the current study was 0.947, indicating strong reliability and validity.

##### Nursing work environment scale

This study used the Nursing Work Environment Scale developed by Ye et al. (2016). The scale includes seven dimensions—career development, leadership and management, nurse-physician relationships, recognition atmosphere, professional autonomy, basic safeguards, and adequate staffing-with a total of 26 items. Example items include: “There are opportunities for career advancement” (career development) and “Nurses participate in hospital internal affairs” (professional autonomy). A 6-point Likert scale was applied, with scores ranging from 1 (strongly disagree) to 6 (strongly agree), resulting in a total score range of 26 to 156. The Cronbach’s α coefficient for this scale in the current study was 0.966, demonstrating excellent reliability and validity.

##### Wong and law’s emotional intelligence scale

This study utilized the WLEIS developed by Wong and Law (2002), which includes four dimensions—self-emotional appraisal, others’ emotional appraisal, emotional regulation, and emotional utilization—comprising a total of 16 items. Example items include: “I have a good understanding of my own emotions” (self-emotional appraisal) and “I am able to control my temper and handle difficulties rationally” (emotional regulation). Responses were rated on a 5-point Likert scale, ranging from 1 (strongly disagree) to 5 (strongly agree), with a total score range of 16–80, where higher scores indicate greater emotional intelligence. The Cronbach’s α coefficient for this scale in the current study was 0.934, demonstrating strong reliability and validity.

### Ethical considerations

The study was conducted according to the guidelines of the Declaration of Helsinki and approved by the Ethics Committee of Hubei Cancer Hospital (LLHBCH2024YN-002). Participants were informed of their right to provide informed consent and voluntarily participate. The trained investigators explained the purpose of the survey and the principle of voluntary withdrawal at any time. All participating nurses provided signed informed consent before the investigation began.

### Data analysis

SPSS 23.0 was used for descriptive analysis, and AMOS 22.0 was used to construct the structural equation models (SEM). Categorical variables were presented as frequencies and proportions, while continuous variables that followed a normal distribution were reported as mean ± standard deviation. Group differences in NWE, EI, and WE across demographic variables were examined using independent samples t-tests or one-way analysis of variance (ANOVA). For ANOVA results that were significant, *post-hoc* comparisons were performed using the Least Significant Difference (LSD) test. Pearson’s correlation coefficient was used to examine the relationships among work engagement, nursing work environment, and emotional intelligence. Structural equation modeling (SEM) was employed to analyze the mediating effect of emotional intelligence between work engagement and the nursing work environment. A total of 2,000 bootstrap resamples were run to test the direct and indirect effects, respectively.

To assess common method variance (CMV), Harman’s single-factor test was conducted using an unrotated exploratory factor analysis. The results indicated that the first factor accounted for 37.12% of the total variance, which is below the 50% threshold, suggesting that CMV was not a serious concern in this study.

Model fit was assessed using several indices: RMSEA (Root Mean Square Error of Approximation), GFI (Goodness of Fit Index), AGFI (Adjusted Goodness of Fit Index), CFI (Comparative Fit Index), PGFI (Parsimonious Goodness-of-Fit Index), and PNFI (Parsimonious Normed Fit Index). A good model fit is indicated by an RMSEA value < 0.05, or at most 0.08, and GFI, AGFI, and CFI values > 0.90, with PGFI and PNFI values exceeding 0.50. The significance level for all tests was set at *p* < 0.05 (Wu, 2020).

## Results

### Data description/demographic characteristics

A total of 523 nurses participated in the study, with data from 510 nurses (97.51%) being analyzed. The majority of participants were female (494, 96.9%), aged 18–35 years (358, 70.2%), married (360, 70.6%), held an intermediate professional title (254, 49.8%), had a bachelor’s degree (436, 85.5%), and were contract employees (506, 75.4%). Regarding night shifts, 344 (67.5%) nurses worked 1–6 night shifts per month, 114 (22.3%) worked more than 6 night shifts per month, and 52 (10.2%) did not work any night shifts. In terms of monthly income, 24 (4.7%) nurses earned less than 3,000 yuan, 294 (57.6%) earned more than 7,000 yuan, and 192 (37.7%) with income between 3000 and 6,999 yuan. In terms of work experience in the HSCT unit, 330 (64.7%) nurses had less than 5 years of experience, 74 (14.5%) had between 5 and 10 years, and 106 (20.8%) had over 10 years of experience.

### Scores

The survey results show that the nursing work environment (120.09 ± 20.86), emotional intelligence (59.6 ± 10.19), and work engagement (60.11 ± 22.55) of HSCT nurses are slightly above average. Significant differences were found in nursing work environment scores based on age (*p* < 0.001), professional title (*p* < 0.001), education level (*p* < 0.05), nature of employment (*p* < 0.05), frequency of night shifts (*p* < 0.001), and monthly income (*p* < 0.001). Emotional intelligence scores also varied according to professional position (*p* < 0.001), years of experience in the HSCT ward, and marital status (*p* < 0.001). Work engagement scores showed significant differences based on age, professional title, professional position, marital status, nature of employment, frequency of night shifts, and monthly income (all *p* < 0.001). *Post hoc* comparisons (LSD) for significant ANOVA results are provided in the table footnotes. Further details can be found in Table 1.

**TABLE 1:** Characteristics of participants and levels of NWE, EI, and WE among nurses in HSCT units.

Variables	Nursing work environment		Emotional intelligence		Work engagement	
	M ± SD	*t*/*F*	M ± SD	*t*/*F*	M ± SD	*t*/*F*
**Gender**						
Male (3.1%)	124.63 ± 20.64	–0.884	56.25 ± 7.24	1.341	61.50 ± 23.52	–0.250
Female (96.9%)	119.94 ± 20.87		59.72 ± 10.26		60.07 ± 22.54	
**Age, year**						
18–35 (70.2%)	124.25 ± 20.62	6.750**	59.09 ± 10.43	1.487	58.01 ± 21.24	8.110**
36–45 (23.5%)	118.67 ± 21.77		60.47 ± 9.57		58.32 ± 25.48	
=46 (6.3%)	109.63 ± 17.91		62.50 ± 7.22		63.56 ± 17.27	
**Professional title**						
Junior(32.5%)	115.00 ± 21.81	8.452**	58.91 ± 10.98	5.550**	56.69 ± 21.08	11.801**
Intermediate (49.8%)	121.65 ± 19.23		59.94 ± 10.38		69.84 ± 22.79	
Senior(17.6%)	125.07 ± 21.79		64.76 ± 8.68		60.08 ± 23.17	
**Working time in HSCT ward, year**						
<5 (64.7%)	120.83 ± 20.70	0.984	59.06 ± 9.90	5.550**	60.37 ± 22.73	8.246**
5–9 (14.5%)	117.00 ± 26.75		59.86 ± 10.35		58.65 ± 25.35	
=10 (20.8%)	119.00 ± 17.24		61.85 ± 11.15		60.34 ± 21.29	
**Education level**						
junior college or below (9.8%)	127.60 ± 22.10	4.427*	59.72 ± 12.92	0.270	66.88 ± 24.40	2.875
Bachelor (85.5%)	118.99 ± 20.53		59.52 ± 9.95		59.17 ± 22.39	
Master or above (4.7%)	124.42 ± 21.22		61.08 ± 8.23		63.17 ± 19.39	
**Marital status**						
Single (27.5%)	122.17 ± 21.28	0.974	60.14 ± 9.91	5.704**	124.63 ± 23.43	8.055**
Married (70.6%)	119.27 ± 20.72		59.12 ± 10.22		57.89 ± 21.97	
Divorce or widowed (2.0%)	120.20 ± 19.58		69.80 ± 7.96		78.80 ± 11.82	
**Nature of employment**						
Authorized strength (23.1%)	131.60 ± 21.67	3.729*	61.76 ± 7.98	1.363	63.37 ± 21.66	4.398**
Contract employee (73%)	120.63 ± 20.79		59.18 ± 10.53		59.84 ± 22.89	
Others (3.9%)	117.87 ± 20.90		59.20 ± 14.95		71.30 ± 25.50	
**Night shift frequency, time/month**						
None (10.2%)	124.98 ± 18.32	8.322**	60.72 ± 9.97	1.812	69.12 ± 22.96	9.216**
1–6 (67.5%)	118.01 ± 21.89		60.01 ± 10.28		59.35 ± 23.06	
6 above (22.3%)	114.00 ± 20.44		56.96 ± 12.47		55.69 ± 23.56	
**Average monthly income, RMB**						
<3,000 yuan (4.7%)	121.05 ± 19.90	5.657**	59.90 ± 9.76	0.521	58.24 ± 21.06	7.630**
3,000–6,999 yuan (37.7%)	120.00 ± 19.68		59.04 ± 13.19		68.68 ± 23.32	
=7,000 yuan (57.6%)	133.42 ± 15.68		61.67 ± 12.76		75.17 ± 24.14	
Nursing work environment	120.09 ± 20.86					
Emotional intelligence	59.61 ± 10.19					
Work engagement	60.11 ± 22.55					

NWE, Nursing Work Environment; EI, Emotional Intelligence; WE, Work Engagement. *p < 0.05; **p < 0.01.

### Model testing

#### Common method variance test

Common method variance was assessed using Harman’s single-factor test (Podsakoff et al., 2003). The results of an unrotated exploratory factor analysis revealed nine factors with eigenvalues greater than 1. The first factor accounted for 37.12% of the total variance, which is below the critical threshold of 50%. This indicates that common method bias was not a serious concern in this study.

#### Correlation analysis of nursing work environment, emotional intelligence, and work engagement

The Average Variance Extracted (AVE) values for all the studied variables ranged from 0.632 to 0.819, demonstrating good convergent validity. Pearson correlation analysis revealed a statistically significant positive correlation between work engagement and the nursing work environment among HSCT nurses (*r* = 0.568, *p* < 0.001). This correlation was consistent across various dimensions of the nursing work environment, including vocational development (*r* = 0.455, *p* < 0.001), leadership and management (*r* = 0.490, *p* < 0.001), doctor-nurse relationships (*r* = 0.485, *p* < 0.001), recognition atmosphere (*r* = 0.563, *p* < 0.001), professional autonomy (*r* = 0.477, *p* < 0.001), basic safeguards (*r* = 0.429, *p* < 0.001), and adequate staffing (*r* = 0.419, *p* < 0.001). Moreover, work engagement exhibited a strong positive correlation with emotional intelligence (*r* = 0.434, *p* < 0.001) and its components: self-emotional monitoring (*r* = 0.194, *p* < 0.001), identification of others’ emotions (*r* = 0.306, *p* < 0.001), emotional regulation (*r* = 0.468, *p* < 0.001), and emotional utilization (*r* = 0.422, *p* < 0.001). Additionally, the nursing work environment was positively correlated with emotional intelligence (*r* = 0.331, *p* < 0.001), as detailed in Table 2.

**TABLE 2:** Correlation matrix for the study variables.

	AVE	*R* ^2^	1	2	3	4	5	6	7	8	9	10	11	12	13	14	15	16	17
1. EI	0.819		1	1	1	1	1	1	1	1	1	1	1	1	1	1	1	1	1
2. SEM			0.748**																
3. IOE			0.805**	0.551**															
4. ER			0.864**	0.478**	0.567**														
5. EU			0.818**	0.429**	0.489**	0.720**													
6. WE	0.632		0.434**	0.194**	0.306**	0.468**	0.422**												
7. Vitality			0.474**	0.239**	0.323**	0.499**	0.457**	0.969**											
8. Dedication			0.407**	0.181**	0.291**	0.436**	0.397**	0.951**	0.911**										
9. Concentration			0.350**	0.125**	0.256**	0.393**	0.343**	0.936**	0.846**	0.822**									
10. NWE	0.671		0.331**	0.202**	0.159**	0.380**	0.322**	0.568**	0.552**	0.542**	0.527**								
11. VD			0.249**	0.149**	0.125**	0.300**	0.221**	0.455**	0.430**	0.443**	0.431**	0.814**							
12. LM			0.303**	0.139**	0.205**	0.310**	0.317**	0.490**	0.477**	0.473**	0.449**	0.854**	0.682**						
13. DNR			0.342**	0.260**	0.215**	0.340**	0.288**	0.485**	0.470**	0.464**	0.452**	0.842**	0.594**	0.704**					
14. RA			0.326**	0.171**	0.144**	0.410**	0.318**	0.563**	0.552**	0.539**	0.516**	0.799**	0.567**	0.583**	0.710**				
15. PA			0.288**	0.199**	0.111*	0.347**	0.266**	0.477**	0.469**	0.439**	0.450**	0.832**	0.556**	0.599**	0.668**	0.755**			
16. BG			0.194**	0.115**	0.047	0.249**	0.209**	0.429**	0.419**	0.417**	0.390**	0.846**	0.597**	0.679**	0.625**	0.594**	0.692**		
17. SM			0.230**	0.139**	0.048	0.281**	0.270**	0.419**	0.414**	0.393**	0.387**	0.811**	0.573**	0.618**	0.586**	0.571**	0.662**	0.761**	
10 and 1 (r^2^)		0.110																	
10 and 6(r^2^)		0.322																	
1 and 6 (r^2^)		0.188																	

AVE, Average Variance Extracted; EI, Emotional Intelligence; SEM; Self-emotion monitoring; IOE, Identify other emotions; ER, Emotional regulation; EU, Emotional utilization; WE; Work engagement; NEW, Nursing work environment; VD, Vocational development; LM, Leadership and management; DNR, Doctor-nurse relationship; RA; Recognize atmosphere; PA; Professional autonomy; BG, Basic guarantee; SM, Sufficient manpower.*p < 0.05, **p < 0.01.

#### Mediating role of emotional intelligence in the association between nursing work environment and work engagement

The indirect effect of emotional intelligence accounted for 24.4% of the total effect, which is considered a moderate to substantial mediation effect in behavioral research contexts (Preacher and Kelley, 2011). This proportion suggests that nearly a quarter of the influence of the nursing work environment on work engagement operates through the enhancement of nurses’ emotional intelligence. In comparison with prior nursing studies testing mediation effects, such as Alinejad et al. where EI mediated 18.6% of the stress–performance relationship (Alinejad et al., 2023), or Ruiz-Frutos et al. where psychological distress mediated about 30% of the environment–engagement relationship (Ruiz-Frutos et al., 2022), our finding falls within a meaningful and clinically relevant range. Within the high-stakes context of HSCT nursing, where emotional and cognitive demands are exceptionally high, the fact that nearly one-quarter of engagement is shaped by the interplay of environmental support and emotional skills underscores the practical significance of fostering both organizational and personal resources.

**FIGURE 1:**
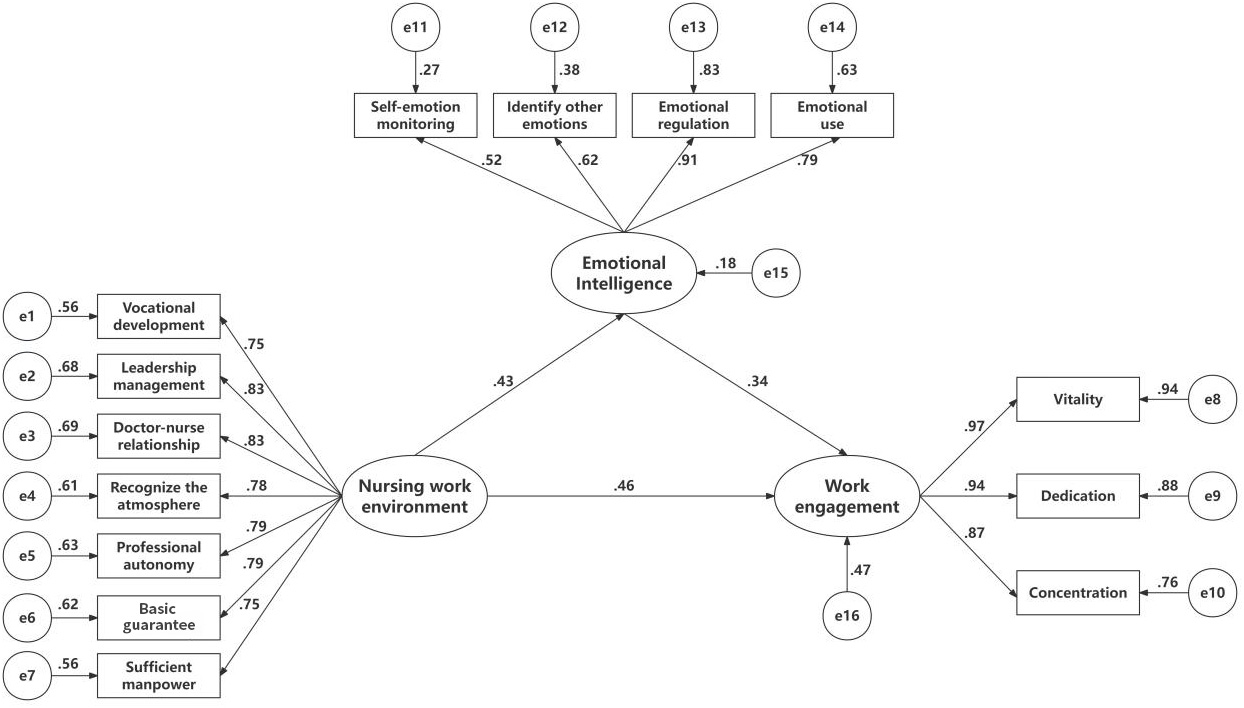
The conceptual model with standardized path coefficients. *p* < 0.0 *p* < 0.01.

The path coefficients and effect sizes presented in Table 3 indicate that the nursing work environment significantly and positively predicted emotional intelligence (β = 0.429, *p* < 0.01). Both emotional intelligence (β = 0.345, *p* < 0.01) and the nursing work environment (β = 0.459, *p* < 0.01) significantly and positively predicted work engagement. Emotional intelligence played a mediating role in the relationship between the nursing work environment and work engagement. As shown in Table 4, the mediating effect accounted for 24.4% (*B* = 0.148, SE Boot = 0.030, 95% CI: 0.095, 0.213). Table 5 presents the comprehensive fit indices of the model, demonstrating favorable alignment with the data. Figure 1 illustrates the standardized path coefficients for each variable within the model.

**TABLE 3:** Path coefficients of the models.

Model path		*β ^a^*	95% CI	*p*
**Direct effect**				
Work engagement **(Y)**	Nursing work environment **(X)**	0.459	[0.348, 0.549]	0.002
Emotional intelligence **(M)**	Nursing work environment **(X)**	0.429	[0.315, 0.536]	0.002
Work engagement (Y)	Emotional intelligence **(M)**	0.345	[0.256, 0.440]	0.001
**Indirect effect**				
Work engagement (Y)	Emotional intelligence **(M)**	0.148	[0.095, 0.213]	0.001

^a^Standardized path coefficient. X represents the independent variable. M represents the mediating variable. Y represents the dependent variable. These bold letters are used to clearly denote the key variables in the hypothesized mediation model, which helps readers quickly, identify the roles of each construct in the path analysis.

**TABLE 4:** Effect analysis of the mediation model.

Model path	*SE* ^a^	S.E.^a^	95% CI	*p*
Total effect	0.607	0.037	[0.529, 0.677]	0.002
Direct effect	0.459	0.050	[0.348, 0.549]	0.002
Indirect effect	0.148	0.030	[0.095, 0.213]	0.001

^a^Standardized path coefficient.

**TABLE 5:** Fit indices of the models.

	Chi-Square	Chi-Square/df	SRMR	RMSEA	GFI	NFI	IFI	TLI	CFI
The model	292.917	4.126	0.044	0.078	0.927	0.946	0.959	0.947	0.959
The standard		≤ 5	≤0.05	≤ 0.08	≥ 0.90	≥0.90	≥ 0.90	≥0.90	≥ 0.90

CFI, comparative fit index; GFI, goodness-of-fit index; IFI, incremental fit index; NFI, normed fit index; RMSEA, root-mean-square error of approximation; SRMR, standardized root-mean-square residual; TLI, Tucker–Lewis index.

Table 3 presents the standardized path coefficients and their significance for the hypothesized paths in the structural equation model. The results show that the direct path from the nursing work environment (NWE) to work engagement (WE) was significant (β = 0.459, *p* < 0.01), providing strong support for Hypothesis H1, indicating that a supportive work environment directly enhances HSCT nurses’ work engagement. Simultaneously, the path from the nursing work environment to emotional intelligence (EI) was also significantly positive (β = 0.429, *p* < 0.01), suggesting that a favorable work environment also helps improve nurses’ personal resource of emotional intelligence. Finally, the path from emotional intelligence to work engagement (β = 0.345, *p* < 0.01) was also significant and positive, strongly supporting Hypothesis H2. The Bootstrap confidence intervals for all these paths did not include zero, further confirming the robustness of these effects.

Table 4 further decomposes the total effect of the nursing work environment on work engagement into direct and indirect effects. The total effect (0.607) represents the overall influence of the work environment on work engagement. This total effect can be decomposed into a direct effect (0.459) and an indirect effect (0.148) mediated by emotional intelligence. Crucially, the Bootstrap 95% confidence interval for the indirect effect [0.095, 0.213] did not include zero, indicating that this mediating effect is statistically significant. Calculation shows that the indirect effect accounts for approximately 24.4% (0.148 / 0.607) of the total effect. This finding confirms Hypothesis H3, that emotional intelligence plays a partial mediating role in the relationship between the nursing work environment and work engagement. This means that the work environment promotes work engagement directly, and also indirectly by fostering nurses’ emotional intelligence, an internal resource.

Table 5 presents the overall goodness-of-fit indices for the final structural equation model. A good model requires the fit indices to meet acceptable standards. As shown in the table, χ2/df = 4.126, which is below the liberal threshold of 5; RMSEA = 0.078, below the cutoff value of 0.08; and indices such as GFI, NFI, IFI, TLI, CFI all exceeded the desired standard of 0.90 (with CFI and IFI reaching 0.959). The SRMR was 0.044, well below the 0.05 standard. Collectively, these indices indicate that our proposed theoretical model, which includes emotional intelligence as a mediator, demonstrates a good fit with the actual data collected from Chinese HSCT nurses. This statistically supports the reasonableness and validity of the theoretical model, meaning the model reasonably represents the true relationships among the variables.

Figure 1 visually presents the validated theoretical model of this study and its standardized path coefficients. The figure clearly distinguishes the direct effect (the path from NWE directly to WE) and the indirect effect (the path from NWE via EI to WE). The coefficients annotated on each path allow for visual comparison of the relative strength of different influence pathways. This figure serves as a visual summary of the statistical results in Tables 3, 4, enabling readers to understand the complex relational mechanisms among the variables at a glance.

## Discussion

This study investigated the relationships between the nursing work environment, emotional intelligence, and work engagement among Chinese HSCT nurses, based on the Job Demands-Resources model. The findings supported all three hypotheses: (1) the nursing work environment was positively associated with work engagement; (2) emotional intelligence was positively associated with work engagement; and (3) emotional intelligence partially mediated the relationship between the nursing work environment and work engagement. These results illuminate the complex interplay between organizational and personal resources in fostering engagement within a high-demand specialty.

Our theoretical propositions find robust support in the specific relationships tested within our structural equation model. The identified pathways not only validate the JD-R model’s core assumptions but also extend our understanding of resource dynamics in high-demand healthcare settings.

### Theoretical interpretation within the JD–R framework

Our findings empirically substantiate the motivational pathway proposed by the JD-R model, wherein job resources (e.g., supportive work environment) foster work engagement. More importantly, we extend the model by demonstrating that job resources also contribute to the development of personal resources—in this case, emotional intelligence. This aligns with the concept of “resource caravan” in JD-R theory, whereby resources tend to accumulate and reinforce one another. Specifically, a supportive NWE provides psychological safety, role clarity, and social support, which in turn create conditions conducive to the development of EI competencies such as emotional regulation, empathy, and interpersonal effectiveness. Emotional intelligence then acts as an amplifying mechanism, enabling nurses to more effectively leverage environmental resources, thereby strengthening the motivational pathway and enhancing work engagement.

### Mechanisms linking nursing work environment to emotional intelligence

The relationship between NWE and EI can be understood through several interconnected mechanisms, deeply rooted in the specific context of HSCT nursing. Firstly, leadership and management styles that emphasize autonomy, participative decision-making, and psychological safety provide nurses with the opportunity to exercise judgment in emotionally charged clinical situations (e.g., managing a patient’s severe anxiety pre-transplant or a family’s grief). This repeated, supported practice is crucial for honing core EI competencies such as self-emotional appraisal and emotional regulation (Wong and Law, 2002; Wei et al., 2018). Secondly, a climate of recognition and respect validates nurses’ emotional labor. In HSCT units, where nurses frequently suppress their own distress to provide stable care, an environment that acknowledges this effort reduces emotional exhaustion and fosters emotional utilization—the ability to harness emotions to facilitate cognitive activities and problem-solving (Freshwater and Stickley, 2004; Zamanzadeh et al., 2013). Thirdly, collaborative nurse-physician relationships and adequate staffing, as key dimensions of NWE, directly reduce cognitive overload and task interruptions. This conservation of cognitive resources frees up the psychological capacity necessary for nurses to engage in reflective emotional processing and demonstrate sustained empathy (others’ emotional appraisal) during prolonged, intense patient interactions (Xia et al., 2016; Lana et al., 2025). Finally, the very nature of the HSCT environment acts as a unique crucible for EI development. The prolonged exposure to patients in isolation, high-stakes clinical outcomes, and complex family dynamics constitute an intense, ongoing training ground. A supportive work environment provides the necessary “scaffolding”—through mentorship, debriefing, and team cohesion—that allows nurses to process these experiences constructively, thereby transforming emotional challenges into opportunities for developing greater emotional awareness and interpersonal effectiveness (Sarabia-Cobo et al., 2017; Sayadi et al., 2019). Thus, a positive NWE does not merely coexist with high EI; It actively cultivates it by providing the safety, resources, and reflective space needed to navigate and grow from the inherent emotional demands of HSCT care.

### Contextualizing the mediation effect in HSCT nursing

The observed 24.4% mediation effect holds substantial clinical and practical significance within the unique ecosystem of HSCT units. Unlike general wards, HSCT nursing is characterized by prolonged patient isolation, high mortality risk, and intense caregiver-family dynamics, leading to exceptional levels of emotional labor and vicarious trauma (Zamanzadeh et al., 2013; Nakamura et al., 2019). In this context, emotional intelligence transcends being a mere “soft skill”; it becomes a critical clinical competency necessary for accurate patient assessment (e.g., detecting subtle signs of depression under immunosuppression), delivering sensitive news, and maintaining therapeutic alliances over extended periods. The finding that nearly a quarter of the work environment’s beneficial impact on engagement flows through EI underscores a powerful leverage point: interventions aimed at enhancing EI are not just additive but are strategic multipliers in high-stress specialties. This proportion suggests that even in environments with resource constraints, targeted investments in developing nurses’ personal emotional resources can yield disproportionately large returns in terms of sustaining engagement, mitigating burnout risk, and fostering the resilience required for long-term retention in this demanding field (Preacher and Kelley, 2011; Wang et al., 2023).

### Direct effects: reinforcing the JD-R model’s core tenets

Our findings on the direct paths strongly reinforce the foundational principles of the JD-R model. First, consistent with prior research (Ruiz-Frutos et al., 2022) and supporting H1, we found that the quality of the nursing work environment directly enhances HSCT nurses’ work engagement. A previous study reported that HSCT patients require approximately 8.9–12.35 h of specialized nursing care daily, with an average of 10.64 hours, and the optimal nurse-to-patient ratio is 1: (–4) (Huang et al., 2014). However, in practical clinical settings, there is often a serious shortage of nursing resources in HSCT wards, leading to long-term overload of nursing staff. Furthermore, the 19 hospitals included in this study required HSCT nurses to rotate between HSCT wards and general hematology wards, requiring continuous adaptation to differing workflows and team dynamics. This repetitive adjustment process compromised HSCT nurses’ ability to maintain focus on patient care, thus reducing their work engagement. Additionally, most of the HSCT nurses in this study were relatively young, with limited HSCT experience and fewer opportunities for on-the-job training and continuing education, which hindered the development of their professional skills, further affecting their work engagement. Moreover, there are no Advanced Practice Nurses (APNs) in HSCT in China, leaving nurses with insufficient understanding of career prospects and preventing them from effectively planning their professional future (Wang et al., 2021). Therefore, nursing administrators should adopt strategies to create a supportive nursing work environment for HSCT nurses. Such strategies could include optimizing human and material resource allocation, promoting collaborative practice within healthcare teams, enhancing nurses’ participation in organizational decision-making, and facilitating professional development, thereby balancing job demands.

We also found that a positive correlation exists between emotional intelligence (EI) and work engagement (WE) (Hypothesis 2). Wong and Law (2002) postulated that individuals with high emotional intelligence are adept at employing various strategies (e.g., prolonging, abbreviating, intensifying, or attenuating emotional experiences) to achieve their objectives, thereby enhancing their levels of work engagement. Xia et al. (2016) reported that 98 nursing items were involved in the bone marrow transplantation ward, and the average total working time for 24-h nursing tasks per patient was 730.86 min. Furthermore, patients undergoing HSCT are prone to mental disorders such as depression, anxiety, distress, and post-traumatic stress disorder, for which nurses are required to provide psychiatric care (Freshwater and Stickley, 2004). Zamanzadeh et al. (2013) described how patients who were seriously ill, had complex medical issues, had been ill for a long time, and were alone in isolated rooms would often confide in nurses. In response, nurses would hide their emotions, suffer in silence, and try to ignore their emotional pain. Additionally, HSCT wards are independent and closed, with nurses facing a heavy workload and fewer opportunities for social activities. Psychological pressure and negative emotions cannot be vented in time, leading to physical and mental fatigue among nurses (Ma et al., 2011). The emotional intelligence of nurses allows them to sense and understand patients’ emotions, which they can then use to manage complex situations related to quality patient care (Freshwater and Stickley, 2004). For HSCT nurses, the higher the level of emotional intelligence, the better they are at managing their own emotions and dealing with others’ problems in positive ways, which promotes work engagement. Therefore, nursing managers should deeply understand the working characteristics of nurses in HSCT wards, implement emotional intelligence training programs (Sarabia-Cobo et al., 2017; Wang et al., 2023), help them explore their inner emotional states, foster emotional cognition and awareness, and encourage them to engage more actively and fully in their work.

The most interesting finding of this study is that the nursing work environment (NWE) for HSCT nurses can directly influence their levels of work engagement, with emotional intelligence (EI) serving as a partial mediator in this relationship. This implies that the nursing work environment also boosts work engagement indirectly, by fostering nurses’ emotional intelligence. The rationale for this mediation effect lies in the ability of HSCT nurses with high emotional intelligence to accurately identify and regulate their emotions, perceive team dynamics, and recognize and address shifts in patients’ emotional states. This finding offers a more complete understanding of the interaction between NWE and EI in work engagement. It suggests that higher emotional intelligence is associated with a more favorable work environment, and that emotional intelligence plays a critical role in fostering a supportive nursing work environment and cultivating harmonious nurse-physician relationships (Wei et al., 2018).

### Theoretical and practical implications

From a practical standpoint, the 24.4% mediation effect provides a compelling, dual-pronged roadmap for nursing management and hospital administration. It argues convincingly that efforts must go beyond improving structural environment factors alone. Firstly, optimizing the organizational resource (NWE) remains foundational and requires: advocating for evidence-based nurse-to-patient ratios (e.g., 1:2–4) in HSCT units to reduce cognitive overload (Huang et al., 2014); developing nurse leaders in transformational styles that empower staff and foster autonomy; establishing clear clinical ladder programs for HSCT specialization (Wang et al., 2021); and implementing structured interprofessional collaboration models. Secondly, and with equal priority, the significant mediating role of EI mandates proactive investment in developing this personal resource. This involves: integrating structured EI training programs into mandatory continuing education, focusing on self-regulation and empathy skills crucial for HSCT care (Sarabia-Cobo et al., 2017; Shrivastava et al., 2022); establishing formal mentorship programs and peer-support groups to facilitate safe emotional processing (Wang et al., 2023); and incorporating reflective practice sessions into routine team meetings to debrief on emotionally challenging cases. By simultaneously strengthening the organizational environment and the individual’s emotional toolkit, healthcare institutions can create a virtuous cycle that maximizes work engagement, safeguards nurse well-being, and ultimately enhances the quality and safety of care for vulnerable HSCT patients.

HSCT nurses frequently work in settings characterized by high cognitive and emotional demands, which can precipitate negative emotional states. The relatively insulated nature of hematopoietic stem cell transplant units, marked by limited social interactions, may hinder the identification of negative emotional experiences among nurses (Zamanzadeh et al., 2013; Sayadi et al., 2019). The high-intensity nursing tasks inherent in this specialty necessitate elevated emotional intelligence for effective management. This capacity contributes to the development of a cohesive team environment and a positive nursing work atmosphere, enhancing work efficiency and the overall quality of patient care. A healthy nursing work environment is conducive to improving nurses’ emotional intelligence. Nurses with high emotional intelligence are more adept at perceiving emotional changes and the communication climate in the environment, utilizing favorable resources, coordinating interpersonal relationships, and thus engaging more fully in their work. Consequently, nursing managers should adjust the working conditions of nurses, implement emotional intelligence and professional training (Ma et al., 2011), allocate organizational resources reasonably, reform leadership practices to empower staff, and stimulate nurses’ sense of responsibility, professionalism, and work enthusiasm to promote work engagement.

### Implications for nursing management

This study confirms the critical associations between NWE, EI, and WE among HSCT nurses in China, with EI serving as a significant mediator accounting for 24.4% of the total effect. These findings compellingly argue for a dual-pronged management strategy: systematically improving the organizational work environment while concurrently enhancing nurses’ emotional intelligence competencies. The following sections detail evidence-based implementation strategies derived from our findings.

#### Enhancing the nursing work environment

Resource Allocation and Staffing: Advocate for and implement evidence-based nurse-to-patient ratios (e.g., 1:2–4) in HSCT units to reduce excessive workload, a key job demand. (1) Leadership and Autonomy: Develop and train nurse leaders in transformational leadership styles that support, empower, and provide autonomy to staff nurses. Involve nurses in unit-level decision-making and policy development. (2) Career Development: Establish clear clinical ladder programs and support pathways for advanced practice roles specific to HSCT nursing to provide career progression and recognition. (3) Fostering Collaboration: Implement structured interprofessional collaboration models (e.g., daily multidisciplinary rounds) to strengthen doctor-nurse relationships and team cohesion.

#### Cultivating emotional intelligence

(1) Structured EI Training: Integrate evidence-based emotional intelligence training programs into mandatory continuing education. These programs should focus on core EI competencies: self-awareness, self-regulation, social awareness, and relationship management. (2) Mentorship and Peer Support: Establish formal mentorship programs pairing experienced HSCT nurses with novices to provide emotional support and guidance. Create peer support groups to facilitate safe sharing and processing of emotional challenges. (3) Reflective Practice: Incorporate facilitated reflective practice sessions into routine team meetings, allowing nurses to debrief on emotionally charged situations and learn from each other’s coping strategies. By strategically investing in these organizational and individual-level interventions, nursing management can create a virtuous cycle where a supportive environment boosts emotional intelligence, which in turn strengthens engagement and performance, ultimately leading to improved nurse retention and patient outcomes in high-stakes HSCT settings.

### Limitations and future research

There are some limitations in our study. First, the results are based on self-reports and should be further explored with additional, more objective measures. Moreover, our study employed a convenient sampling method due to the large number of hospitals in China and limited research funding. In future studies, we will use a stratified random sampling method, which would significantly enhance the robustness of the research, particularly in large-scale investigations. Additionally, the study focused solely on a single work resource (the nursing work environment) and one individual resource (emotional intelligence) in relation to work engagement. Other factors, which may also influence work engagement through mediating effects, were not considered. Future research should explore the impact of multiple resources and demands on work engagement, as well as their interrelationships. Finally, multi-center and longitudinal research designs should be pursued to elucidate the developmental trajectories of HSCT nurses, providing empirical evidence for the formulation of individualized interventions at different stages of their professional development. Furthermore, as data were collected from multiple hospitals, a clustering effect at the hospital level may exist due to shared institutional contexts (e.g., management systems, organizational culture). This potential bias was not controlled for in the current analysis and should be addressed in future studies using multilevel modeling techniques.

## Conclusion

In conclusion, this study demonstrates that among HSCT nurses, work engagement is jointly shaped by the nursing work environment and emotional intelligence, with EI serving as a meaningful partial mediator. These findings extend the JD-R model by illustrating a specific resource pathway in which organizational resources foster personal resources, which in turn amplify engagement. For nursing leaders, this implies that dual-focused interventions—optimizing the work environment while concurrently building emotional intelligence—are essential for enhancing nurse engagement and resilience in high-demand settings such as hematopoietic stem cell transplantation.

## Data Availability

The original contributions presented in the study are included in the article/supplementary material, further inquiries can be directed to the corresponding author.
